# Cesarian sections in women with multiple sclerosis: A Canadian prospective pregnancy study

**DOI:** 10.1177/20552173241285546

**Published:** 2024-10-08

**Authors:** Dessa Sadovnick, Maria Criscuoli, Irene Yee, Robert Carruthers, Virginia Devonshire, Penelope Smyth, Kristen M Krysko

**Affiliations:** Department of Medical Genetics, University of British Columbia, Vancouver, Canada; Department of Medicine, Division of Neurology, 8166 University of British Columbia, Vancouver, Canada; Department of Medical Genetics, University of British Columbia, Vancouver, Canada; Department of Medicine, Division of Neurology, 8166 University of British Columbia, Vancouver, Canada; Department of Medical Genetics, University of British Columbia, Vancouver, Canada; Department of Medicine, Division of Neurology, 8166 University of British Columbia, Vancouver, Canada; Department of Medicine, Division of Neurology, 8166 University of British Columbia, Vancouver, Canada; Department of Medicine (Neurology), University of Alberta, Edmonton, Canada; BARLO MS Centre, Division of Neurology, Department of Medicine, 10071 St Michael's Hospital, University of Toronto, and Li Ka Shing Knowledge Institute, Toronto, Canada

**Keywords:** Multiple sclerosis, pregnancy, cesarian sections

## Abstract

**Background:**

An increasing number of women with multiple sclerosis (wMS) are considering pregnancy. Prior studies suggest increased rate of elective cesarian sections (C-sections) in wMS.

**Methods:**

The Canadian Multiple Sclerosis Pregnancy Study (CANPREG-MS) is a prospective study on pregnant wMS. This report shows comparisons between (i) CANPREG-MS wMS delivered by C-section and the general population and (ii) C-section and vaginal deliveries in this study cohort.

**Results:**

CANPREG-MS has resulted in 170 deliveries with 63 by C-section. The proportion with C-sections in CANPREG-MS (37.1%) was significantly higher than that for the Canadian population (28%) (*p* = .0085). The majority (66.7%) of C-sections were not planned, and typically were performed for obstetrical indications. C-sections were performed at an earlier gestational age than vaginal deliveries, although birthweight did not differ by mode of delivery in wMS. MS relapses (3.2%) and pseudo-relapses (3.2%) were rare in the first month after C-section deliveries, regardless of disease modifying therapy decisions during gestation and postpartum.

**Conclusions:**

C-sections were more common in wMS than the general population, but few were because of maternal MS. CANPREG-MS provides informative data for pregnancies in wMS with well-managed and relatively mild disease. This information is helpful to obstetrical and MS healthcare providers.

## Introduction

Multiple sclerosis (MS), an inflammatory demyelinating and neurodegenerative condition of the central nervous system, is the most common cause of nontraumatic neurologic disability in young adults. MS preferentially affects women, with the clinical onset usually occurring during the reproductive years. Revised diagnostic criteria^1^ allowing earlier diagnosis and the early use of disease modifying therapies (DMTs),^2^ have resulted in an increasing number of women with MS (wMS) considering pregnancy.^3^ In Canada and the United States, the current prevalence of MS approaches 290/100,000 population with up to 75% being genetically female.^4^ Family planning, pregnancy, and methods of delivery are frequent topics of discussion between wMS and healthcare professionals.^5^

The focus of this paper is to provide what we believe to be the first prospective data on cesarian section deliveries (C-sections) for wMS. Obstetrical care providers (family physicians, obstetricians, midwives) often label wMS as having “high risk pregnancies” only because of the diagnosis.^5,6^ This could lead to recommendations for C-sections in the absence of obstetrical needs.^6^ In a recent nationwide Danish study, elective nonemergency C-sections had a higher rate in wMS compared to the general population.^7^ Obstetrical care providers may be relatively inexperienced in caring for wMS, thus limiting their experience with pregnancy and delivery. Even the advice of MS neurologists is inconsistent with some believing C-sections are beneficial to avoid the stress and fatigue of labor and delivery while still recognizing general C-section maternal and infant risks.^8^ In contrast, others feel that unless MS symptoms or obstetrical issues provide a specific need for a C-section, vaginal delivery is the safest preferred option.

The objective of this paper is to examine C-section deliveries in a well-described cohort of wMS who participated in the Canadian Multiple Sclerosis Pregnancy Study (CANPREG-MS).^9^ The C-section rate in this study was compared to general Canadian population data available from the Canadian Institute for Health Information.^10^ The group of CANPREG-MS vaginal deliveries was also used for comparisons with the C-section group. Data on maternal MS relapses/pseudo-relapses within one month of C-section delivery by DMT exposure/resumption are presented here. Short-term outcomes are described as they relate to mode of delivery, and it is beyond the scope of this paper to discuss events after the first month postpartum as data collection is in progress.

## Methods

CANPREG-MS is a prospective Canadian pregnancy study to develop a Canada-wide prospective registry of women with MS who are either trying to become pregnant and/or have become pregnant.^9^ CANPREG-MS follows enrolled wMS from trying to conceive until up to 12 months postpartum. Resultant livebirths are followed to age 12 months with regard to health and developmental milestones. Specific details have been published elsewhere.^9^ This paper focuses on C-section deliveries by participating wMS.

CANPREG-MS inclusion criteria were as follows: (1) a confirmed diagnosis of MS aged 18 and over; (2) wMS who are pregnant or actively planning a pregnancy; and (3) Canadian residents. Specific to this C-section study, additional inclusion criteria were that a participant had: (1) a pregnancy that resulted in a livebirth; (2) completed their entry initial interview prior to delivery and (3) completed the one month postpartum CANPREG-MS interview.

Participants were self-referred and the study was run centrally at the University of British Columbia (UBC). Ethics approval was given by the UBC Clinical Research Board and Vancouver Coastal Health Research Institute. All participants provided written informed consent to participate in the study and to allow CANPREG-MS to access their medical health records to confirm the diagnosis of MS.

Enrolled pregnant wMS were followed at set times during pregnancy and after delivery.^9^ Telephone interviews used standardized ethics approved questionnaires which allow free-text comments. Thus, it was possible to obtain more details than possible using population-based health administrative databases.

Data regarding mode of delivery (C-section or vaginal delivery, with or without vacuum and forceps) as well as pregnancy and infant outcomes were collected.

CANPREG-MS participants were asked in detail about MS relapses/pseudo-relapses and DMT exposures at least one-month preconception, during gestation and postpartum.

For this specific study, the abbreviation “PP” only refers to the one-month postpartum period. An MS relapse was defined as new or worsening neurologic symptoms lasting ≥ 24 hours without concurrent fever, infection, or other metabolic issues. A pseudo-relapse was temporary worsening of neurologic symptoms with concurrent fever, infection or metabolic issues.^11^ In contrast to MS relapses, MS pseudo-relapses do not result from new inflammatory disease activity, and thus do not show radiographic evidence of active demyelination on MRI, although it was beyond the scope of this study to require MRI confirmation of relapses. Ambiguous cases were reviewed by one of the neurologist authors. CANPREG-MS^9^ used the Patient Determined Disease Steps tool (PDDS)^12^ to assess disability.

DMTs are referred to using generic and trade names to reflect the information conveyed by participants and/or their available medical records.

For analyses, CANPREG-MS proportions with C-section, preterm birth and postterm birth in percentages were compared with the corresponding Canadian rates using chi-square tests. Birthweight was summarized with mean and standard deviation (SD), and the mean was compared to the Canadian average using a *t*-test. General population Canadian rates were obtained from the Canadian Institute for Health Information^10^ (C-sections), the Canadian Preterm Birth Network^13^ (preterm births), the Public Health Agency of Canada^14^ (postterm births), and Statistics Canada^15^ (birthweight). Two-sided tests were used and *p*-value of <.05 was considered statistically significant.

Data are presented as median and interquartile range (IQR) for age at pregnancy onset, gestational age and birthweight. Differences between wMS having “vaginal” deliveries and “C-section” deliveries were assessed using Mann–Whitney tests (two-tailed with significance level of 5%) for numerical variables and chi-square test for the categorical disability variable (PDDS < 2 (0 = no disability; 1 = mild disability) at pregnancy). Other data including infant outcomes, as well as MS relapses and pseudo-relapses are presented descriptively.

## Results

There were a total of 172 deliveries among 159 wMS in CANPREG-MS. Thirteen wMS had two livebirths each during the study period. Two wMS were subsequently excluded from analyses. One had major neurological complications (confidentiality prevents further details here). The other was excluded as she delivered after agreeing to enroll in CANPREG-MS but delivered early and prior to completing the initial study interview. Thus, the final study group consisted of 170 deliveries (two twin births) resulting in 172 livebirths from 157 wMS. Of these, 63 were C-sections, including four women who had two C-section livebirths each. Overall characteristics of these women by mode of delivery are given in Table 1.

**Table 1.** Characteristics of CANPREG-MS wMS by mode of delivery.

	C-section delivery *n* = 63 pregnancies with 65 livebirths^a^	Vaginal delivery *n* = 107 pregnancies with 107 livebirths	Statistics
Age at pregnancy onset, median years (IQR^b^)	32.54 (5.46)	32.54 (4.79)	Mann–Whitney test, *p* = .64
Gestational age, median weeks (IQR)	39 (2.00)	40 (2.07)	Mann–Whitney test, *p* = .018
Birth weight, median grams (IQR)	3345.24 (759.18)^c^	3345.24 (564.39)^d^	Mann–Whitney test, *p* = .87
PDDS^e^ at pregnancy^f^, n (%)			
0	33 (52.38%)	66 (61.68%)	
1	16 (25.40%)	21 (19.63%)	
2	10 (15.87%)	12 (11.21%)	
3	3 (4.76%)	4 (3.74%)	
4	0	4 (3.74%)	
5	1 (1.59%)	0	
PDDS < 2, *n* (%)	49 (77.78%)	87 (81.31%)	Chi-square test, *p* = .58

CANPREG-MS: Canadian Multiple Sclerosis Pregnancy Study; wMS: women with multiple sclerosis.

^a^ 63 pregnancy events resulted in 65 livebirths (two sets of twins).

^b^ IQR = interquartile range.

^c^ Excluding two livebirths from C-section delivery with unknown birth weight.

^d^ Excluding five livebirths from vaginal delivery with unknown birth weight.

^e^ PDDS = Patient Determined Disease Steps.

^f^ PDDS at the first interview during pregnancy.

PDDS rated disability at pregnancy was relatively mild (PDDS < 2) for most wMS who had C-sections (49/63). Ten had moderate disability (PDDS = 2) and four had gait disabilities (PDDS = 3 or 5).

There was no evidence of cognitive issues, as discussed elsewhere.^9^

The C-section rate in CANPREG-MS (37.1%) was significantly higher than for the general Canadian population (28% in 2015–2016)^10^—chi-square = 6.92, degree of freedom (*df*) = 1, *p* = .0085 (95% confidence interval (CI): [30.16%, 44.53%]).

Of the 63 C-sections, 21 were planned (see Table 2). The two twin pregnancies delivered by C-section were initially to be vaginal deliveries but complications resulted in C-sections. Two C-sections were preplanned only because of the maternal MS. There were nine unplanned C-sections due to difficult labor (these wMS did state that the doctors were concerned about the fatigue and stress of long labor (ranging from 18 h to 60 h)), but it could not be determined whether MS was really the factor contributing to deciding on a C-section.

**Table 2.** Indications for C-section deliveries.

Indication for C-section	Number of deliveries	%
Planned C-section (*n* = 21)		
Prior C-section	6	28.57
Pregnancy complications	5	23.81
Fetal position	3	14.29
Slow/restricted growth	2	9.52
MS	2	9.52
Other	3	14.29
Unplanned C-section (*n* = 42)		
Difficult labor	9	21.43
Abnormal fetal heart rate	8	19.05
Fetal position	4	9.52
Umbilical cord abnormalities	4	9.52
Pregnancy complications	4	9.52
Fetal macrosomia	3	7.14
Slow/restricted growth	2	4.76
Multiple gestation	2	4.76
Other	6	14.29

MS: multiple sclerosis.

### CANPREG-MS compared to the general Canadian population

Preterm birth is defined as birth before 37 weeks’ gestation and occurs in about 8% of pregnancies in Canada.^13^ There were 16 preterm births in CANPREG-MS of which eight were delivered by C-section. There was no significant difference when the preterm birth proportion for Canadian wMS (9.30%) was compared with that of the Canadian general population (8%)^13^—chi-square = 0.396, *df* = 1, *p* = .53 (95% CI [5.81%, 14.58%]).

Postterm births are defined as birth at 42 weeks’ gestation or later.^14^ There were five postterm births in CANPREG-MS (2.91%), two of which were delivered by C-section, which is higher compared to 0.61% in the 2010 Canadian general population^14^—chi-square = 14.97, *df* = 1, *p* = .00011 (95% CI [1.25%, 6.62%]).

There was no difference in the mean birthweight of CANPREG-MS infants (3339.63 grams; *SD* = 544.59) compared to the 2016 Canadian average (3367 grams)^15^—*t* = −0.646, *df* = 164, *p* = .52 (95% CI [3255.92, 3423.34] grams).

### C-sections compared to vaginal deliveries within CANPREG-Ms

Median gestational age was statistically different by mode of delivery. The gestational age of the newborns delivered by vaginal deliveries (median = 40 weeks; IQR = 2.07) was older compared to C-section deliveries (median = 39 weeks; IQR = 2.00) (*p* = .018). There were no statistically significant differences between mode of delivery groups regarding age at conception, proportion with PDDS < 2, and birthweight of their newborn (see Table 1 for details). Forceps and/or vacuum were used in 12/107 vaginal deliveries (11.21%) compared to 3/63 (4.8%) C-sections (Table 3).

**Table 3.** Instrumental delivery.

Delivery type	Instrument used during delivery		Number of deliveries	%
	
	Vacuum	Forceps		
Vaginal (*n* = 107)	Yes	No	5	4.67
	No	Yes	3	2.80
	Yes	Yes	4	3.74
C-section (*n* = 63)	Yes	No	3	4.76

### Infant outcomes

No major health issues or birth defects were identified for the 65 newborns delivered by C-section within PP, except for the eight preterm infants. One of the preterm infants with birthweight <2000 grams had feeding problems and was in neonatal intensive care unit (NICU) for 12 days. Not unexpectedly for multiples, four infants from two twin births were in the NICU because of prematurity and weight. The remaining three preterm singleton infants stayed in NICU between 1 and 3 days. Although final data collection for many in the dataset is ongoing, these eight infants were in fact studied up to 12 months after delivery and all were meeting developmental milestones.

### MS treatment and outcomes

Regardless of DMT usage preconception or PP, MS relapses and pseudo-relapses were rare after C-section deliveries, with two (3.2%) having MS relapses and two (3.2%) having pseudo-relapses PP (Tables 4 and 5, and Supplemental Table S1).

**Table 4.** MS relapses and pseudo-relapses by DMT exposure at conception and after delivery (up to 1 month postpartum (PP)) among C-section deliveries.

	DMT^a^ naïve (*n* = 13)	Completed recommended DMT washout before conception (*n* = 31)	DMT exposure at conception—washout period shorter than FDA recommendation (*n* = 6)	DMT use at conception (*n* = 13)
Age at pregnancy onset, median years (IQR^b^)	34.04 (2.17)	32.00 (5.39)	31.41 (2.44)	32.21 (5.21)
Gestational age, median weeks (IQR)	38.14 (2.15)	39.00 (1.36)	39.00 (1.50)	39.14 (2.00)
DMT at conception, n (%)				
None	13 (100)	31 (100)	0 (0)	0 (0)
Glatiramer acetate	0 (0)	0 (0)	0 (0)	8 (61.5)
Dimethyl fumarate	0 (0)	0 (0)	0 (0)	1 (7.7)
Natalizumab	0 (0)	0 (0)	0 (0)	4 (30.8)
Ocrelizumab	0 (0)	0 (0)	6 (100)	0 (0)
DMT restart/start after delivery, *n* (%)				
None	12 (92.3)	27 (87.1)	2 (33.3)	6 (46.2)
Glatiramer acetate	0 (0)	0 (0)	0 (0)	3 (23.1)
Dimethyl fumarate	0 (0)	1 (3.2)	0 (0)	0 (0)
Natalizumab	0 (0)	1 (3.2)	0 (0)	3 (23.1)
Ocrelizumab	1 (7.7)	2 (6.5)	4 (66.7)	1 (7.7)
Relapses^c^ PP, *n* (%)	0 (0)	1 (3.2)	0 (0)	1 (7.7)
Pseudo-relapses^c^ PP, *n* (%)	1 (7.7)	0 (0)	0 (0)	1 (7.7)
PDDS^d^ PP, *n* (%)				
0	6 (46.15%)	16 (51.61%)	4 (66.67%)	5 (38.46%)
1	6 (46.15%)	8 (25.81%)	2 (33.33%)	5 (38.46%)
2	1 (7.70%)	3 (9.68%)	0	2 (15.39%)
3	0	2 (6.45%)	0	0
4	0	2 (6.45%)	0	1 (7.69%)

^a^ DMT = disease modifying therapy.

^b^ IQR = interquartile range.

^c^ None of the cases with PP relapses or pseudo-relapses had started DMT after delivery at the time of the event.

^d^ PDDS = Patient Determined Disease Steps.

**Table 5.** DMT status at conception and at 1-month postpartum (PP) among C-section deliveries.

DMT^a^ status at conception	DMT status at PP	*n*	%
DMT naïve (*n* = 13)			
None	None	12	19.05
None	Ocrelizumab	1	1.59
Completed recommended DMT washout before conception (*n* = 31)			
None	None	27	42.86
None	Dimethyl fumarate	1	1.59
None	Natalizumab	1	1.59
None	Ocrelizumab	2	3.17
DMT exposure at conception—washout period shorter than FDA recommendation (*n* = 6)			
Ocrelizumab	None	2	3.17
Ocrelizumab	Ocrelizumab	4	6.35
DMT use at conception (*n* = 13)			
Natalizumab	Natalizumab	3	4.76
Natalizumab	Ocrelizumab	1	1.59
Glatiramer acetate	Glatiramer acetate	3	4.76
Glatiramer acetate	None	5	7.94
Dimethyl fumarate	None	1	1.59

^a^ DMT = disease modifying therapy.

Thirteen participants were DMT naïve up to and including at conception and during gestation. One woman had a pseudo-relapse 1 week after her C-section with epidural anesthesia, but her PDDS did not change (PDDS = 1) and she recovered spontaneously without any treatment. One of these women received her first dose of Ocrevus (ocrelizumab) 3 weeks after delivery but had no relapse/pseudo-relapse.

Thirteen women were using a DMT at conception. Of these, 12 pregnancies were planned and one was unplanned. Four of the 13 were on Tysabri (natalizumab) and all continued DMT while pregnant, but infusions were reduced from every 4 weeks to extended interval dosing at every 6 weeks. One woman had her last infusion at 34 weeks’ gestation but resumed right after delivery. None had a relapse/pseudo-relapse PP and PDDS was <2. One woman switched from natalizumab to ocrelizumab after delivery by choice.

There were eight women on glatiramer acetate (six Copaxone; two Glatect) at conception; two continued Copaxone throughout pregnancy and PP. Neither had a pseudo-relapse/relapse PP. The remaining six women discontinued glatiramer acetate after a confirmed pregnancy, the latest at 7 weeks’ gestation. Of these, one had a pseudo-relapse PP and another had a relapse that began during the third trimester. One woman was on Tecfidera (dimethyl fumarate) but stopped after pregnancy confirmation. She did not resume any DMT PP, with no postpartum relapse/pseudo-relapse.

Overall, 31 wMS completed a washout preconception for their specific DMT based on their neurologists’ recommendations. Four resumed DMT PP which included dimethyl fumarate (*n* = 1); natalizumab (*n* = 1); ocrelizumab (*n *= 2), and none had a relapse/pseudo-relapse PP. The remaining 27 wMS did not resume a DMT by PP, and one woman reported a relapse PP.

## Discussion

Overall, within this cohort, the proportion of C-sections (37.1%) was higher than the overall rate for the general population. Importantly, the majority (66.7%) of C-sections were unplanned, with typical obstetrical indications in the majority of cases. Canadian obstetrical healthcare providers did not appear to extensively preplan C-section only based on the mother having MS, as has been suggested elsewhere.^6^ However, MS may have had an impact on more rapid decisions to deliver by C-section if labor was prolonged, and in two cases a C-section was planned due to MS diagnosis alone. It is important to note that this cohort was relatively well-managed and with low levels of MS-related disability, which may differ from other study populations using different sources of ascertainment.

There was also a higher rate of postterm births in wMS (2.91% compared to 0.61% in the Canadian general population), but the sample size was small. Although the literature^7^ suggests that wMS have smaller babies, this was not the case in CANPREG-MS with a similar mean birthweight to the Canadian average. There was also similar birthweight for C-section and vaginal deliveries, despite earlier delivery in terms of weeks’ gestation for C-sections compared to vaginal deliveries, as would be expected in the case of planned C-sections.

Compared to other recent work evaluating C-sections in MS (e.g. Houtchens et al.,^3^ De Giglio et al.,^6^ and Andersen et al.^7^), this study is unique as it was designed as prospective. The main benefit of this approach as opposed to a retrospective approach was avoidance of recall bias and the ability to ask targeted questions about MS symptoms/relapses, although there is the potential for selection bias based on participants who chose to participate. In addition, all subjects had a confirmed MS diagnosis by criteria prior to pregnancy (thus excluding women who had onset or diagnosis during gestation). Data collection was over a short time period (2018–2022), thus minimizing temporal changes in MS diagnosis and/or treatment that often occurs in longitudinal population-based studies, e.g. the Danish study covered the period 1 January 1997 to 31 December 2016.^7^

Overall, MS relapses (3.2%) and pseudo-relapses (3.2%) were rare in the one month after C-section deliveries. Relapses were defined clinically, without requirement for objective change on neurologic examination, or confirmation of new MRI activity. Thus, it is possible some pseudo-relapses may have been considered relapses if there was no concurrent fever, infection or metabolic disturbance. However, as the absolute numbers of reported relapses were low, this is a minor caveat in the current study.

Various DMT strategies were used in the wMS who had C-sections, with some conceiving before starting DMT, others stopping DMT in advance of pregnancy, and others continuing DMT. FDA guidelines for various DMTs are not always optimum in real life where risks to the wMS must be evaluated and balanced with the potential risk to the pregnancy (e.g. pregnancy loss, birth defects). This was well illustrated here for ocrelizumab. FDA^16^ recommends a 6-month washout prior to conception, but this guideline was not strictly followed in the C-section group. A 6-month washout is recommended by some neurologists^17^ while more recently others suggest trying to conceive 1–3 months after an infusion for women with active MS.^5,18^ A European publication recommended women receiving regular ocrelizumab for MS should wait for 3 months before trying to conceive.^19^ DMT washouts as described by the FDA may not be the best option in the real world where the risk to the mother has to be compared to the risk to the unborn child, as discussed in an editorial.^20^ Real world studies such as CANPREG-MS thus provide critical information. Relapses and pseudo-relapses were rare in this study regardless of DMT strategy.

There are limitations to this study. Participation was voluntary.^9^ This likely biased the study toward healthier, well-managed wMS, and planned pregnancies rather than unplanned pregnancies or wMS with highly active, disabling disease and/or cognitive impairment. Attempts have been internationally made to identify a cohort of pregnant women with secondary progressive MS but with small numbers and varying geographic access to medical care, this has been unsuccessful to date. Secondly, information was provided by interview with participants and it was beyond the scope of this study to obtain medical confirmation of all the data. However, this is no different than using population-based registries and the additional information from participants’ comments provided clarity and further insight. Further, there is a potential for confounding, as adjustment for potential confounders when comparing with Canadian population-based data was not possible. Further studies with a control group allowing for adjustment for potential confounders such as age are required to confirm associations. Finally, the current study focuses on outcomes at the 1-month post-partum interview. It is recognized that that not all birth defects/congenital malformations/developmental delays are identified close to birth, and the first 3 months postpartum is also a high risk period for MS relapses. Further follow up is underway to 12 months after delivery of all livebirths.

In summary, C-sections were more common in wMS than the general Canadian population, although these were generally done for obstetrical indications rather than maternal MS. Infant and maternal status at 1-month PP after C-sections were reassuring thus providing positive information for wMS who are well-managed and relatively well during their reproductive years. This paper provides real world data that adds to our previously published work on the road to conception^21^ and early pregnancy losses.^22^ This information will be helpful to wMS and their obstetrical and MS healthcare providers.

## Supplemental Material

sj-docx-1-mso-10.1177_20552173241285546 - Supplemental material for Cesarian sections in women with multiple sclerosis: A Canadian prospective pregnancy study Supplemental material, sj-docx-1-mso-10.1177_20552173241285546 for Cesarian sections in women with multiple sclerosis: A Canadian prospective pregnancy study by Dessa Sadovnick, Maria Criscuoli, Irene Yee, Robert Carruthers, Virginia Devonshire, Penelope Smyth and Kristen M Krysko in Multiple Sclerosis Journal – Experimental, Translational and Clinical
