# Supplementary material for: Cesarian sections in women with multiple sclerosis: A Canadian prospective pregnancy study
Source: Mult Scler J Exp Transl Clin. 2024 Oct 8;10(4):20552173241285546. doi: 10.1177/20552173241285546 (PMC11459475; doi:10.1177/20552173241285546)
Supplement: sj-docx-1-mso-10.1177_20552173241285546 - Supplemental material for Cesarian sections in women with multiple sclerosis: A Canadian prospective pregnancy study [file sj-docx-1-mso-10.1177_20552173241285546.docx]

Supplementary Table S1: MS relapses and pseudo-relapses by DMT exposure at conception and after delivery (up to 1 month postpartum (PP)) amongst C-section deliveries, additional details of cases

| **DMT^a^ at conception** | **Restart/start DMT after delivery** | **Number of relapse/pseudo-relapse** | **PDDS^b^ at 1 month PP** | **Number of pregnancies** |
| --- | --- | --- | --- | --- |
| **DMT Naïve (n=13)** | | | | |
| Naïve | No | 0 | 0 | n=5 |
| Naïve | No | 0 | 1 | n=5 |
| Naïve | No | 0 | 2 | n=1 |
| Naïve | No | 1 pseudo-relapse 1 week after delivery (had epidural) | 1 | n=1 |
| Naïve | Yes-Ocrevus  (3 weeks after delivery) | 0 | 0 | n=1 |
| **Completed recommended DMT washout (n=31)** | | | | |
| No | No | 0 | 0 | n=13 |
| No | No | 0 | 1 | n=7 |
| No | No | 0 | 2 | n=2 |
| No | No | 0 | 3 | n=2 |
| No | No | 0 | 4 | n=2 |
| No | No | 1 relapse; almost fully recovered at PP | 2 | n=1 |
| No | Yes-Ocrevus  (2 weeks after delivery) | 0 | 0 | n=1 |
| No | Yes-Ocrevus  (PP) | 0 | 0 | n=1 |
| No | Yes-Tecfidera  (PP) | 0 | 0 | n=1 |
| No | Yes-Tysabri  (2 weeks after delivery) | 0 | 1 | n=1 |
| **DMT exposure at conception - washout period shorter than FDA recommendation (n=6)** | | | | |
| Yes-Ocrevus (< 6 months washout) | No | 0 | 0 | n=1 |
| Yes-Ocrevus (< 6 months washout) | No | 0 | 1 | n=1 |
| Yes-Ocrevus (< 6 months washout) | Yes-Ocrevus  (PP) | 0 | 0 | n=2 |
| Yes-Ocrevus (< 6 months washout) | Yes-Ocrevus  (PP) | 0 | 1 | n=1 |
| Yes-Ocrevus (< 6 months washout) | Yes-Ocrevus  (2 weeks after delivery) | 0 | 0 | n=1 |
| **DMT use at conception (n=13)** | | | | |
| Yes-Copaxone | Yes-Copaxone  (never discontinued) | 0 | 4 | n=1 |
| Yes-Copaxone | Yes-Copaxone  (never discontinued) | 0 | 0 | n=1 |
| Yes-Copaxone: stopped after a positive pregnancy test | No | 1 pseudo-relapse at time of C-section: fully recovered by PP | 1 | n=1 |
| Yes-Copaxone: stopped after a positive pregnancy test | No | 1 relapse started at last trimester of pregnancy with tingling in upper legs and fatigue that continued after the 1^st^ month postpartum | 2 | n=1 |
| Yes-Copaxone: stopped after a positive pregnancy test | No | 0 | 0 | n=2 |
| Yes-Glatect: stopped 7 weeks into pregnancy | No | 0 | 0 | n=1 |
| Yes-Glatect: stopped after a positive pregnancy test | Yes-Glatect  (2 weeks after delivery) | 0 | 1 | n=1 |
| Yes-Tecfidera; stopped after a positive pregnancy test | No | 0 | 1 | n=1 |
| Yes-Tysabri (stopped at week 35) | Yes-Ocrevus  (PP) | 0 | 1 | n=1 |
| Yes-Tysabri | Yes-Tysabri  (never discontinued) | 0 | 0 | n=1 |
| Yes-Tysabri (every 6 weeks since she found out about pregnancy) | Yes-Tysabri  (never discontinued) | 0 | 1 | n=1 |
| Yes-Tysabri (every 6 weeks; stopped at week 34) | Yes-Tysabri  (PP) | 0 | 2 | n=1 |

^a^DMT= disease modifying therapy; trade name is used to simplify the table for obstetrical care providers and wMS: Copaxone (glatiramer acetate), Glatect (glatiramer acetate), Tysabri (natalizumab), Ocrevus (ocrelizumab), Tecfidera (dimethyl fumarate)

^b^PDDS= Patient Determined Disease Steps
